# Distribution of cannabinoid synthase genes in non-Cannabis organisms

**DOI:** 10.1186/s42238-019-0008-7

**Published:** 2019-08-05

**Authors:** Niranjan Aryal, Debbie Figueroa Orellana, Jamal Bouie

**Affiliations:** Socal Cannabis Science Research Group (ScCSRG), Los Angeles, CA USA

**Keywords:** Cannabinoids, Tetrahydrocannabinoids, Cannabinoid synthase, *Morus notabilis*, Cannabinoid synthesis pathway, *Cannabis sativa*

## Abstract

The discovery of phytocannabinoid synthesizing enzymes, tetrahydrocannabinolic acid synthase (THCAs) and cannabidiolic acid synthase (CBDAs) was a breakthrough in Cannabis research. However, their evolutionary aspects and distribution across organisms has not been adequately studied. We searched for THCAs and CBDAs genes in organisms other than Cannabis plants using the database available in NCBI. Both cannabinoid synthases seem to be widely distributed in the plant kingdom. Of several complete or partial sequences of cannabinoid synthases-likes, CBDAs-like from *Morus notabilis* matched closely to CsCBDAs and CsTHCAs. When amino acid sequences of CsTHCAs, CsCBDAs and MnCBDAs-like were compared to each other, and to the motif database stored in Expasy, all three proteins contained the FAD_PCMH (PCMH-type FAD-binding) domain indicating the conservation of this domain in cannabinoid synthases. Apart from FAD binding, Berberine Bridge Enzyme (BBE-likes), which catalyzes the synthesis of isoquinoline alkaloids in many plants such as mulberry, poplas and citrus, were the other most closely related enzymes to CsTHCAs and CsCBDAs. We also searched for THCAs and CBDAs in fungal and bacterial kingdom but could not find any notably similar sequence. However, partial mRNA from FAD binding enzyme from *Trametes versicolor* and 6-hydroxy D nicotine oxidase from *Aspergillus saccharolyticus* matched the CsTHCA sequence and a partial mRNA from a hypothetical protein in *Pneumocystis carinii* was the most closely matched fungal enzyme to the CsCBDA. Our database search showed that *Morus notabilis* from mulberry family could be the candidate plant for further studies. Comparative transcriptomic and metabolomic studies for mulberry and Cannabis plants could provide a much clear concepts on the co-evolution of these syanthases. Moreover, the understanding of cannabinoid synthesis pathway is still evolving, in-depth bioinformatics and functional analysis of the enzymes involved are required for pharmaceutical research and industrial advancement.

**Electronic supplementary material:**

The online version of this article (10.1186/s42238-019-0008-7) contains supplementary material, which is available to authorized users.

## Background

The domestication and use of Cannabis plants for several applications started much earlier than the Christian era (Russo 2007). Since then, such plants have been used for various purposes such as fiber, textiles, and papers. Evidence of human consumption of Cannabis products for both medicinal and recreational values can be dated as back as 2500 BC (De Petrocellis et al. 2000). More recently, scientists have been looking for chemical constituents present in Cannabis plants that are believed to be responsible for psychoactive effect in the human brain. The complete knowledge of the biosynthesis and working mechanisms of these chemical constituents, termed as cannabinoids, has not yet been obtained. Theories of these cannabinoids working together with other secondary metabolites, such as terpenoids and flavonoids, have recently been purposed by researchers (Atwal et al. 2018).

**Fig. 1:**
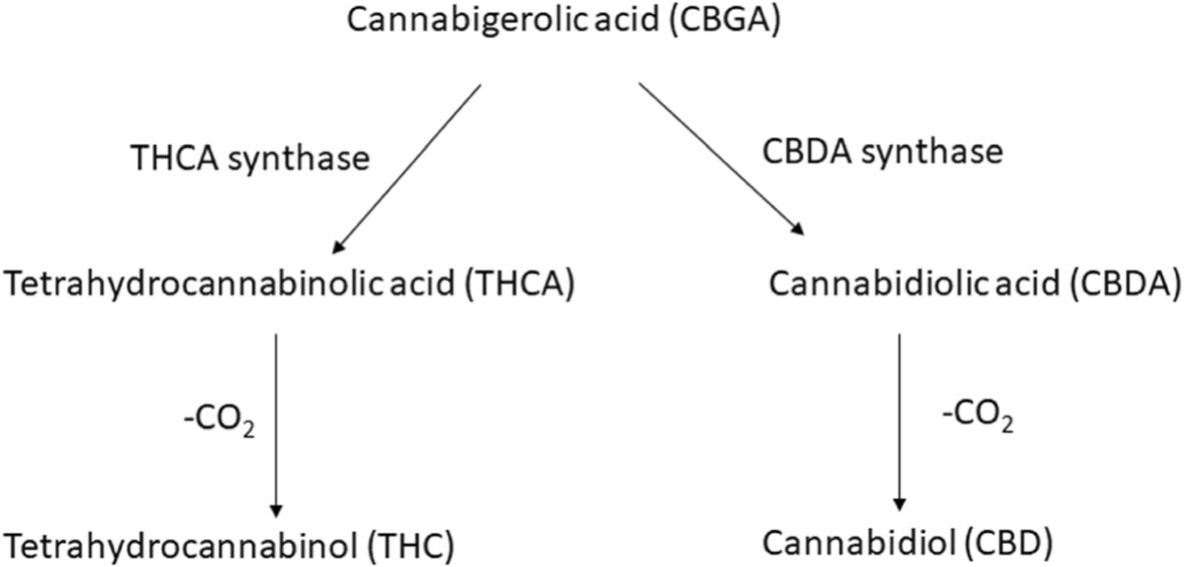
Synthesis of cannabinoids, THC and CBD, from CBGA

More than 480 chemical compounds are produced by the *Cannabis sativa*, of which cannabinoids constitute more than 100 (Pollastro et al. 2018). THC and CBD are the main cannabinoids that have gained most of the attention. Both compounds are formed by the non-enzymatic decarboxylation of their non-active acidic forms; THCA and CBDA respectively (Onofri et al. 2015). THC is a psychoactive compound that binds to the endocannabinoid receptor of vertebrates (Ameri 1999), while CBD is non-toxic. THCA synthase is the enzyme responsible for the production of THCA; CBDA synthase is the enzyme responsible for the production of CBDA. Both enzymes compete for the common substrate Cannabigerolic acid (CBGA) (Fig. 1) (Onofri et al. 2015). Cannabis plants are classified into two types, Marijuana and Hemp, based on the amount of THC and CBD they produce. Marijuana produces high THC and low CBD amounts, while hemp produces high CBD and low THC amounts (Sawler et al. 2015). Published studies show that the transcript level of THCAs and CBDAs might be the determining factor in the synthesis of THC/CBD; however, the mechanism for variations in expression of these genes is still not completely explained. Two theories, the mutual exclusiveness and the close linkage of THCAs and CBDAs, are being debated (de Meijer et al. 2003; Kojoma et al. 2006). A recently published study proposes a different model which mentions that CBDAs and THCAs are not isoforms at an otherwise equivalent locus (Laverty et al. 2018).

Despite the long history of traditional breeding and selection of different Cannabis strains, research at the molecular or genetic level is still at onset. The draft of whole genome and transcriptome for marijuana and for hemp were published in 2011; the article compared thegenetic differences among these two types (van Bakel et al. 2011). A recent study on heterogenicity of THCAs and CBDAs in different strains of *Cannabis sativa* found the SNPs in these transcripts which could have caused the difference in chemical phenotype. The study also proposed CBDAs as the ancestral enzyme of both enzymes (Onofri et al. 2015). For a better understanding of the evolution of cannabinoid genes, a wider exploration of these enzymes in the whole plant kingdom and other organisms is required. In this paper, we searched for THCAs and CBDAs in organisms other than *Cannabis sativa*.

## Methods

All the nucleotide sequences used in the analysis were obtained from NCBI. mRNA sequences for CsTHCAs, CsCBDAs and MnCBDAs-like used for this study are listed in the Additional file 1. CsTHCAs and CsCBDAs were each blasted against the plants (taxid:3913), fungi (taxid:4751), algae (taxid:2864) and bacteria (taxid:2), excluding *Cannabis sativa* to ensure the wider coverage in the given taxa. Closely matched subject sequences were selected from the blasts hit and their FASTA files were downloaded from the genebank. MEGA7 was then used to build the neighbor joining phylogenetic tree (Kumar et al. 2016; Tamura et al. 2004; Saitou and Nei 1987). The sequences were also aligned using the online portal for Clustwal W alignment (https://www.ebi.ac.uk/Tools/msa/clustalo/). Expasy (https://prosite.expasy.org) was used for alignment and motif scanning of the protein sequences.

## Results

### THCAs and CBDAs in plant kingdom

**Fig. 2:**
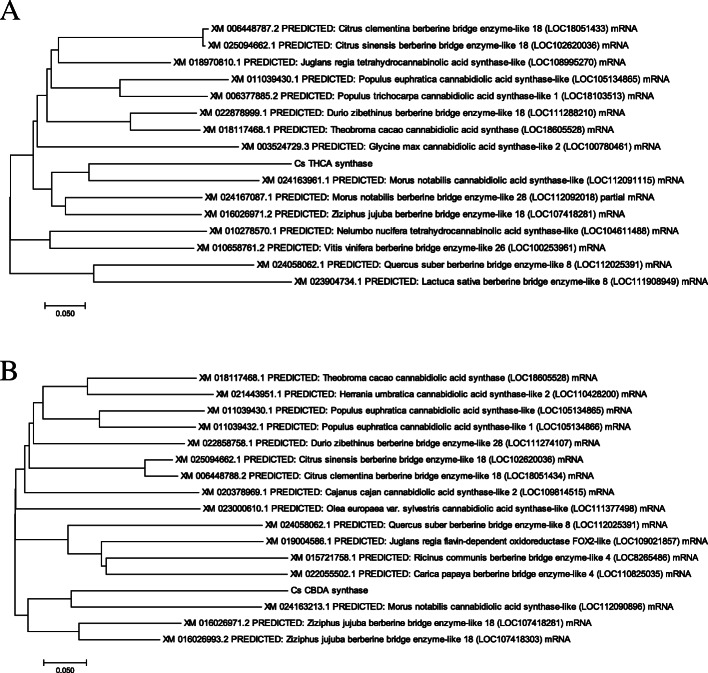
Neighbor joining phylogenetic tree for the **a** CsTHCAs and **b** CsCBDAs with the closest enzymes from plant kingdom

**Fig. 3:**
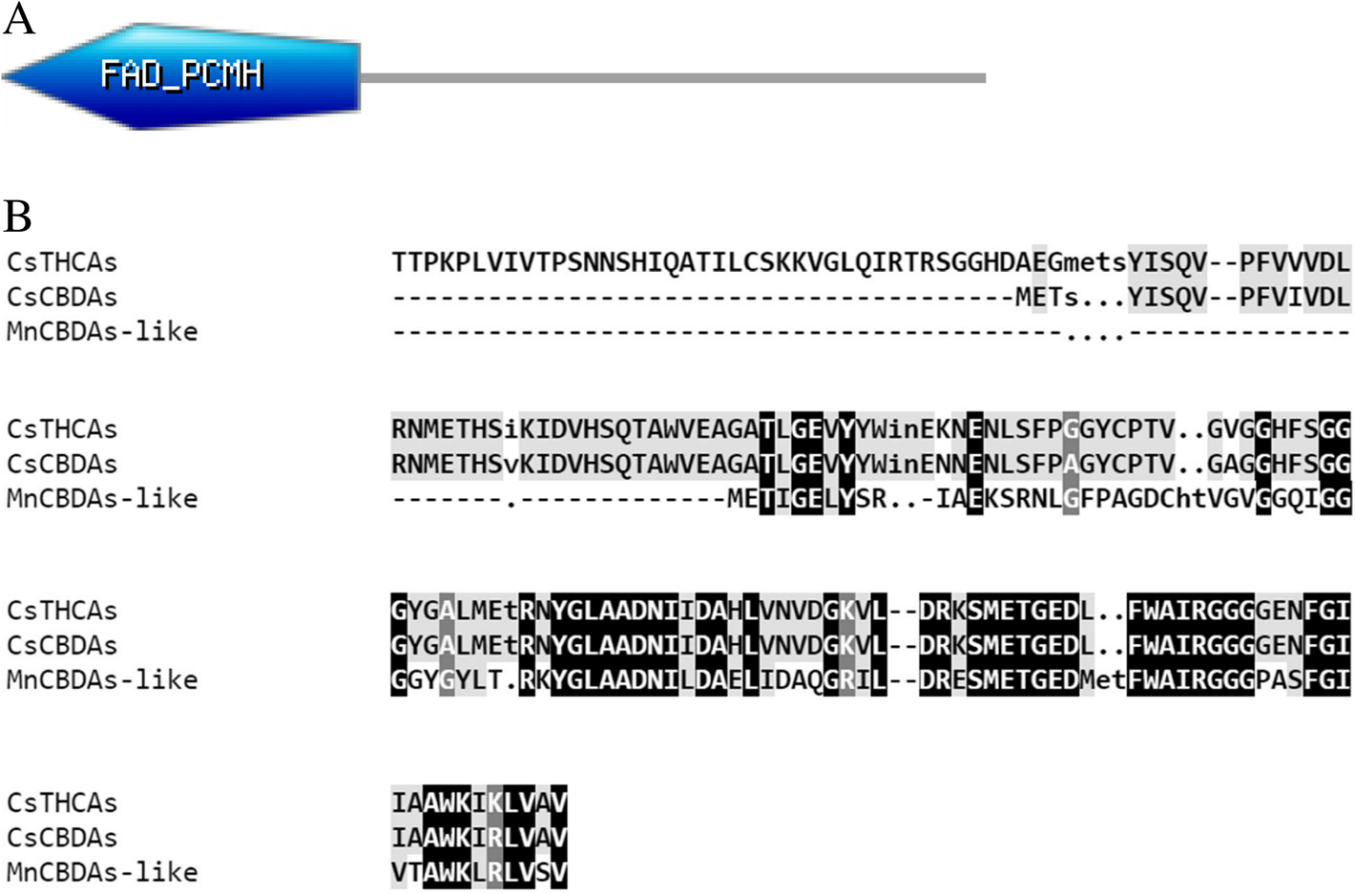
**a** FAD_PCMH domain in the CsTHCAs, CsCBDAs and MnCBDAs-like obtained from scanning of protein in Prosite Expasy. **b** Alignment of aminoacid sequences representing the FAD_PCMH domain in three proteins

We searched for the THCAs and CBDAs related enzymes in the plant kingdom using the NCBI nucleotide blast (https://blast.ncbi.nlm.nih.gov/Blast.cgi). Cannabidiolic acid synthase like from *Morus notabilis* was the most closely related enzyme for both THCAs and CBDAs synthases from *Cannabis sativa* (Fig. 2a and b). The mRNA sequence of CBDAs-like from *Morus notabilis* was closer to CsCBDAs (66% sequence identity for 99% query cover) than CsTHCAs (66% sequence identity with 77% query cover). Comparing the CsTHCAs, CsCBDAs and MnCBDAs-like aminoacid sequences to each other and to the motif database stored in prosite Expasy (https://prosite.expasy.org/), all three proteins contain the FAD_PCMH (PCMH-type FAD-binding) domain (Fig. 3a). The amino acid length for FAD_PCMH domain in the CsTHCAs was longer than that of CsCBDAs and MnCBDAs-like (Fig. 3b). *Morus notabilis* is a species from the Mulberry family. Silkworms, which produce silk, feed on the leaves of Mulberry plant (He et al. 2013). The plant also produces edible fruits. A draft of whole genome sequences of the plant Morus notabilis was published in 2013 (He et al. 2013). Interestingly, *Cannabis sativa* was most closely aligned to the *Morus notabilis* in a phylogenetic tree that was produced by using single copy genes from *M. notabilis, T. cacao, A. thaliana, P. trichocarpa, S. lycopersicum, V. vinifera, P. bretschneideri, M. domestica, P. persica, F. vesca, C. sativa, M. truncatula and O. sativa* (He et al. 2013). However, we could not find any evidence of the cannabinoid compounds from the Mulberry tree. Also, there are no studies that discuss the presence of a cannabinoid synthesis pathway in the plant.

Berberine Bridge Enzyme (BBE-likes) were the other most closely related enzymes to CsTHCAs and CsCBDAs. BBE enzymes catalyzes the synthesis of isoquinoline alkaloids which are secondary metabolites produced by several plants such as Mulberry, Poplar, and citrus. THCAs like was aligned *Juglans regia* was aligned to CsTHCAs. However, the sequence identity was less (72% identity for 56% query cover).

### THCA and CBDA synthases in Fungi

**Fig. 4:**
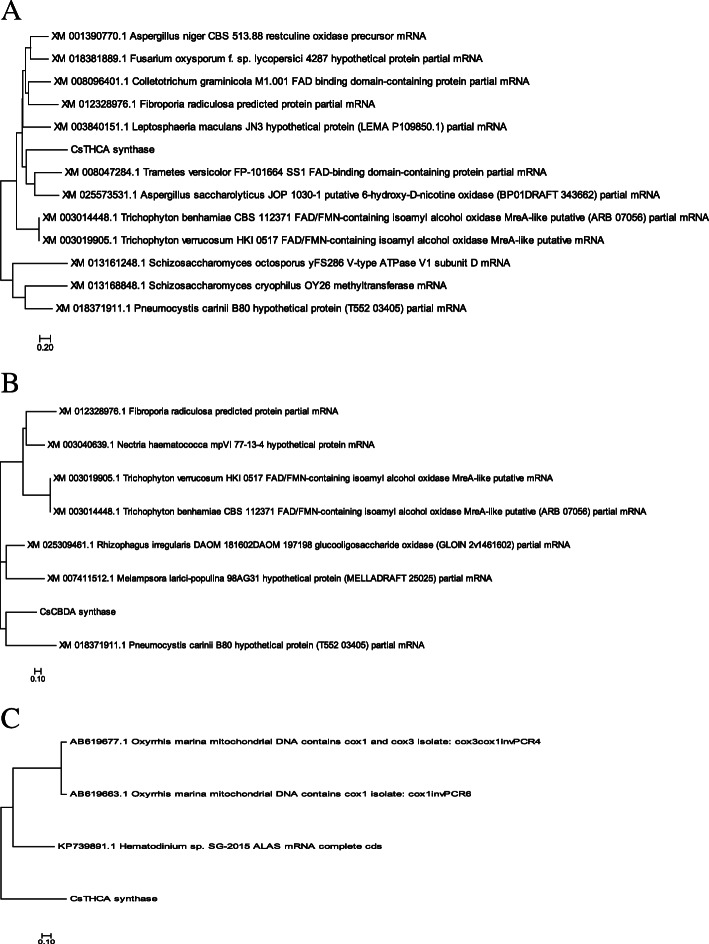
Neighbor joining phylogenetic tree for **a** CsTHCAs and closest enzyme from fungi **b** CsCBDAs and closest enzymes from fungi **c** CsCTHCAs and closest enzymes from algae

We also looked for the THCAs and CBDAs like enzymes in fungi and algae. Both THCAs and CBDAs were aligned closely to the FAD-binding enzymes from fungi. Partial mRNA from FAD binding enzyme from *Trametes versicolor* and 6-hydroxy D nicotine oxidase from *Aspergillus saccharolyticus* matched the CsTHCA sequence (Fig. 4a). CsCBDA was also aligned to the FAD binding enzymes from fungi (Fig. 4b). A partial mRNA from a hypothetical protein in *Pneumocystis carinii* was the most closely matched fungal enzyme to the CsCBDA. Any evidence for the synthesis of cannabinoid compounds or the presence of complete pathway for cannabinoid synthesis has not been reported from fungi. When the match for CsTHCA was searched for in the algae, three enzymes were distantly aligned to CsTHCA sequences (Fig. 4c). For bacterium, there were no notable sequence matches for either CsTHCA or CsCBDA.

## Discussion

As the world is progressing on the Cannabis legalization, scientific research on medical values and genetic aspects are of high demand. Though the Cannabis plant has been bred and selected for desired level of THC/CBD ratio and terpenoid flavor, its underlying genetic basis has not been adequately understood.. Complete knowledge on the evolution and distribution of the enzymes associated with cannabinoid biosynthesis is still lacking. Much larger and rigorous studies on different aspects including agronomy, biochemistry and genetics are required for advancing the knowledge on evolution and phylogeny of the Cannabis plant and its metabolomics.

Cannabis plant shows specific and contrasting natural selection characteristics. Secondary metabolites are usually negatively selected during the domestication process of plants. However, cannabinoids in Cannabis plants seems to have been enhanced via breeding and selection (van Bakel et al. 2011). Studies on many unique properties that make Cannabis a compelling plant could lead us to the next level of understanding the plant’s pharmacology.

Biosynthesis of unique compounds such as cannabinoids, terpenes and flavonoids in non-Cannabis organisms is gaining much interest as an industry or the research. Attempts for synthesizing THC and CBD in yeast and microorganisms are actively researched (Carvalho et al. 2017). Heterologous production of isoprenoid, a large family of secondary metabolites, has been successful in both yeast and *E.coli* (Paddon and Keasling 2014). Recently, complete heterologous production of natural and unnatural cannabinoids in yeast has been reported. The biosynthesis required introduction of a complete hexanoyl-CoA pathway from multiple organisms and cannabinoid synthases from *Cannabis sativa* (Luo et al. 2019). An organism already having endogenous primary pathways could be a better candidate for the production of cannabinoids and other secondary metabolites native to *Cannabis sativa*..

## Conclusions

This paper discusses the probable presence and expression of THCAs and CBDAs like enzymes across the plant kingdom and in fungi and algae. Having CBDAs-like enzyme, *Morus notabilis* from Mulberry family could be the candidate plant for further studies. More in-depth structural bioinformatics analysis and invitro expression of these proteins are required before making any further conclusion.

## Additional file

Additional file 1: Data 1. mRNA sequences for CsTHCAs, CsCBDAs and MnCBDAs-like. The sequences were used to search against the database in NCBI and create the neighbor-joining trees in MEGA. (DOCX 15 kb)

## Data Availability

All data was obtained from NCBI.

## Abbreviations

CBDAs: Cannabidiolic acid synthase
CBGA: Cannabigerolic acid
Cs: *Cannabis sativa*
THCAs: Tetrahydrocannabinolic acid synthase
