# Supplementary material for: Distribution of cannabinoid synthase genes in non-Cannabis organisms
Source: J Cannabis Res. 2019 Aug 5;1:8. doi: 10.1186/s42238-019-0008-7 (PMC7819296; doi:10.1186/s42238-019-0008-7)
Supplement: Supplementary file 1 — Data 1. mRNA sequences for CsTHCAs, CsCBDAs and MnCBDAs-like. The sequences were used to search against the database in NCBI and create the neighbor-joining trees in MEGA. (DOCX 15 kb) [file 42238_2019_8_MOESM1_ESM.docx]

Distribution of cannabinoid synthase genes in non-Cannabis organisms

Additional Data

Additional file 1: Data 1: mRNA sequences for CsTHCAs, CsCBDAs and MnCBDAs-like. The sequences were used to search against the database in NCBI and create the neighbor-joining trees in MEGA.

>CcTHCAs

AAAAAAATCATTAGGACTGAAGAAAAATGAATTGCTCAGCATTTTCCTTTTGGTTTGTTTGCAAAATAATATTTTTCTTTCTCTCATTCCATATCCAAATTTCAATAGCTAATCCTCGAGAAAACTTCCTTAAATGCTTCTCAAAACATATTCCCAACAATGTAGCAAATCCAAAACTCGTATACACTCAACACGACCAATTGTATATGTCTATCCTGAATTCGACAATACAAAATCTTAGATTCATCTCTGATACAACCCCAAAACCACTCGTTATTGTCACTCCTTCAAATAACTCCCATATCCAAGCAACTATTTTATGCTCTAAGAAAGTTGGCTTGCAGATTCGAACTCGAAGCGGTGGCCATGATGCTGAGGGTATGTCCTACATATCTCAAGTCCCATTTGTTGTAGTAGACTTGAGAAACATGCATTCGATCAAAATAGATGTTCATAGCCAAACTGCGTGGGTTGAAGCCGGAGCTACCCTTGGAGAAGTTTATTATTGGATCAATGAGAAGAATGAGAATCTTAGTTTTCCTGGTGGGTATTGCCCTACTGTTGGCGTAGGTGGACACTTTAGTGGAGGAGGCTATGGAGCATTGATGCGAAATTATGGCCTTGCGGCTGATAATATTATTGATGCACACTTAGTCAATGTTGATGGAAAAGTTCTAGATCGAAAATCCATGGGAGAAGATCTGTTTTGGGCTATACGTGGTGGTGGAGGAGAAAACTTTGGAATCATTGCAGCATGGAAAATCAAACTGGTTGCTGTCCCATCAAAGTCTACTATATTCAGTGTTAAAAAGAACATGGAGATACATGGGCTTGTCAAGTTATTTAACAAATGGCAAAATATTGCTTACAAGTATGACAAAGATTTAGTACTCATGACTCACTTCATAACAAAGAATATTACAGATAATCATGGGAAGAATAAGACTACAGTACATGGTTACTTCTCTTCAATTTTTCATGGTGGAGTGGATAGTCTAGTCGACTTGATGAACAAGAGCTTTCCTGAGTTGGGTATTAAAAAAACTGATTGCAAAGAATTTAGCTGGATTGATACAACCATCTTCTACAGTGGTGTTGTAAATTTTAACACTGCTAATTTTAAAAAGGAAATTTTGCTTGATAGATCAGCTGGGAAGAAGACGGCTTTCTCAATTAAGTTAGACTATGTTAAGAAACCAATTCCAGAAACTGCAATGGTCAAAATTTTGGAAAAATTATATGAAGAAGATGTAGGAGCTGGGATGTATGTGTTGTACCCTTACGGTGGTATAATGGAGGAGATTTCAGAATCAGCAATTCCATTCCCTCATCGAGCTGGAATAATGTATGAACTTTGGTACACTGCTTCCTGGGAGAAGCAAGAAGATAATGAAAAGCATATAAACTGGGTTCGAAGTGTTTATAATTTTACGACTCCTTATGTGTCCCAAAATCCAAGATTGGCGTATCTCAATTATAGGGACCTTGATTTAGGAAAAACTAATCATGCGAGTCCTAATAATTACACACAAGCACGTATTTGGGGTGAAAAGTATTTTGGTAAAAATTTTAACAGGTTAGTTAAGGTGAAAACTAAAGTTGATCCCAATAATTTTTTTAGAAACGAACAAAGTATCCCACCTCTTCCACCGCATCATCATTAATTATCTTTAAATAGATATATTTCCCTTATCAATTAGTTAATCATTATACCATACATACATTTATTGTATATAGTTTATCTACTCATATTATGTATGCTCCCAAGTATGAAAATCTACATTAGAACTGTGTAGACAATCATAAGATATATTTAATAAAATAAATTGTCTTTCTTATTTCAATAGCAAATAAAATAATATTATTTTAAAAAAAAAAAAAAAAA

>CsCBDAs

CCCAAAACCACTTGTTATCATCACTCCTTTAAATGTCTCCCATATCCAAGGCACTATTCTATGCTCCAAGAAAGTTGGCTTGCAGATTCGAACTCGAAGCGGTGGTCATGATGCTGAGGGCATGTCCTACATATCTCAAGTCCCATTTGTTATAGTAGACTTGAGAAACATGCATTCGGTCAAAATAGATGTTCATAGCCAAACTGCATGGGTTGAAGCCGGAGCTACCCTTGGAGAAGTTTATTATTGGATCAATGAGAACAATGAGAATCTTAGTTTTCCTGCTGGGTACTGCCCTACTGTTGGCGCGGGTGGACACTTTAGTGGAGGAGGCTATGGAGCATTGATGCGAAATTATGGCCTCGCGGCTGATAATATCATTGATGCGCACTTAGTCAATGTTGATGGAAAAGTTTTAGATCGAAAATCCATGGGGGAAGATTTGTTTTGGGCTATACGTGGTGGTGGAGGAGAAAACTTTGGAATCATTGCAGCGTGGAAAATTAGACTTGTTGCTGTCCCATCAATGTCTACTATATTCAGTGTTAAAAAGAACATGGAGATACATGAGCTTGTCAAGTTAGTTAACAAATGGCAAAATATTGCTTACATGTATGAAAAAGAATTATTACTCTTTACTCACTTTATAACCAGGAATATTACAGATAATCAAGGGAAGAATAAGACAACAATACACAGTTACTTCTCCTCCATTTTCCATGGTGGAGTGGATAGTCTAGTCGACTTGATGAACAAGAGCTTTCCTGAATTGGGTATTAAAAAAACAGATTGCAAACAGTTGAGCTGGATTGATACTATCATCTTCTACAGTGGTGTTGTAAATTACAACACAACTTATTTTAAAAAAGAAATTTTGCTTGATAGATCAGGTGGGCGGAAGGCGGCTTTCTCGATTAAGTTAGACTATGTTAAGAAACCGATTCCAGAAACCGCAATGGTCACAATTTTGGAAAAATTATATGAAGAAGATGTAGGAGTTGGGATGTTTGTGTTTTACCCTTATGGTGGTATAATGGATGAGATTTCAGAATCAGCAATTCCATTCCCTCATCGAGCTGGAATCATGTATGAAATTTGGTACATAGCTTCATGGGAGAAGCAAGAAGATAATGAAAAGCATATAAACTGGATTCGGAATGTTTATAATTTCACGACTCCTTATGTGTCCCAAAATCCAAGAATGGCGTATCTCAATTATAGGGACCTTGATTTAGGAAAAACTAATTTCGAGAGTCCTAATAATTACACACAAGCACGTATTTGGGGTGAAAAGTATTTTGGTAAAAATTTTAATAGGTTAGTAAAAGTAAAAACCAAGGTTGATCCCGATAATTTCTTTAGAAACGAACAAAGCATCCCACCTCTTC

>MnCBDAs-like CCCAAAACCATTTGTTATCATCACGCCATTTCACGTCTCCCACGTCCAAGCCACTGTTTTCTGCTCCAGAAAACACGGCATGCAAATCAGAACCCGAAGTGGTGGCCATGATTATGAGGGCCTTTCTTATGTGTCCAGTGTCTCGTTTGTCATAATTGATCTGAGAAACCTTAATTTGATCAACGTAGACGTGAAGAGTAAATCTGCTTGGGTTCAAGCTGGAGCTACCATTGGGGAACTTTATTATAGGATTGCTGAAAAAAGTGAAAATCTCGCCTTCCCAGCCGGTGATTGCCCTGGTGTGGGTATTGGTGGACAGATCGGTGGAGGAGGCTATGGCTATTTGGCACGAAAATACGGCCTCGCAGCTGATAATGTTCTTGACGCGGAAGTAATCGATGTTAAAGGAAGAATTCTTGATAGGAAATCTATGGGTGAAGATTTGTTCTGGGCCATACGTGGTGGCGGACCAGCAAGCTTTGGAATCGTTCTTTCATGGAAACTTCAACTATTTCCTGTTCCATCGATAGTGACTGTGTTTGATGTAAGTAGGAACATGGAAGACAATACAACAAAGAAGTTCGTTCATCAATGGCAACGCCGTGCTGACAAAGTTGATGAGGATCTATCAATCTATGGCAGGTTCCAAACTGAGAGTTCTGTTGATAATGAAGGCAATAAAAAAATTGTGCTGCGTGCTTCTTTCCGCGCAACATACCATGGCGGTGTGGATAAGCTCCTTCAATTGATGCAAAAGGAGTTTCCTGAATTGGGTTTGCTAAGACAAGAGTGCCATGAAATGAAATGGGCCGAAACCTTTCTCTTTCTCAATTATCTCAGAAATGGAGAATCCTTAGATGTTCTACTTAATAGGACATCTACTATCAAGTCGGCATTCAAAGCAAAATCTGACTTTGTGAAAAAACCTATTTCAGATGACATATTCGAAAAAATGTTGGGAAAGTTGTACGAAGTGGTAGGAAGTGCTTTTATTGATCTATTCCCTTTAGGAGGGAAAATGAACGAGATTTCGGAATCCGCAATCCCGTTCCCATACCGAGCTGGAAACCTCTACAACATTCATTATTTAGTTGGATGGCAAGAAGATGGGAATATTACAACGTCCAAAAAGCTTGTGGATTGGATAAGAAAGTTTTACAATTACATGACTCCTTATGTGTCAAAAAATCCGAGGGCTACGTATCTCAATTTCAGAGACCTTGACATCGGCACTAATAATAATGACGACACCACAAGTTCTTATAAAAATATTGCACGGGCAAGGATTTGGGGTACTAAGTATTTTAAAGATAATTTCTATAAATTAGTTTACGTAAAGACTATAATTGATCCAACTAATTTCTTTAGAAATGAACAAAGCATCCCACCTCTTC
